# Does anthropometric status at 6 months predict the over-dispersion of malaria infections in children aged 6–18 months? A prospective cohort study

**DOI:** 10.1186/s12936-019-2778-y

**Published:** 2019-04-22

**Authors:** Jaden Bendabenda, Noel Patson, Lotta Hallamaa, Ulla Ashorn, Kathryn G. Dewey, Per Ashorn, Kenneth Maleta

**Affiliations:** 1 Department of Public Health, School of Public Health, University of Malawi College of Medicine, Mahatma Gandhi Road, Private Bag 360, Blantyre 3, Malawi; 2 Faculty of Medicine and Life Sciences, Center for Child Health Research, University of Tampere, Tampere, Finland; 3 Department of Nutrition, University of California, Davis, Davis, CA USA; 4 School of Public Health, University of the Witwatersrand, Johannesburg, South Africa

**Keywords:** Children, iLiNS studies, Infections, Malaria, Over-dispersion, Stunting, Undernutrition, Wasting

## Abstract

**Background:**

In malaria-endemic settings, a small proportion of children suffer repeated malaria infections, contributing to most of the malaria cases, yet underlying factors are not fully understood. This study was aimed to determine whether undernutrition predicts this over-dispersion of malaria infections in children aged 6–18 months in settings of high malaria and undernutrition prevalence.

**Methods:**

Prospective cohort study, conducted in Mangochi, Malawi. Six-months-old infants were enrolled and had length-for-age z-scores (LAZ), weight-for-age z-scores (WAZ), and weight-for-length z-scores (WLZ) assessed. Data were collected for ‘presumed’, clinical, and rapid diagnostic test (RDT)-confirmed malaria until 18 months. Malaria microscopy was done at 6 and 18 months. Negative binomial regression was used for malaria incidence and modified Poisson regression for malaria prevalence.

**Results:**

Of the 2723 children enrolled, 2561 (94%) had anthropometry and malaria data. The mean (standard deviation [SD]) of LAZ, WAZ, and WLZ at 6 months were − 1.4 (1.1), − 0.7 (1.2), and 0.3 (1.1), respectively. The mean (SD) incidences of ‘presumed’, clinical, and RDT-confirmed malaria from 6 to 18 months were: 1.1 (1.6), 0.4 (0.8), and 1.3 (2.0) episodes/year, respectively. Prevalence of malaria parasitaemia was 4.8% at 6 months and 9.6% at 18 months. Higher WLZ at 6 months was associated with lower prevalence of malaria parasitaemia at 18 months (prevalence ratio [PR] = 0.80, 95% confidence interval [CI] 0.67 to 0.94, p = 0.007), but not with incidences of ‘presumed’ malaria (incidence rate ratio [IRR] = 0.97, 95% CI 0.92 to 1.02, p = 0.190), clinical malaria (IRR = 1.03, 95% CI 0.94 to 1.12, p = 0.571), RDT-confirmed malaria (IRR = 1.00, 95% CI 0.94 to 1.06, p = 0.950). LAZ and WAZ at 6 months were not associated with malaria outcomes. Household assets, maternal education, and food insecurity were significantly associated with malaria. There were significant variations in hospital-diagnosed malaria by study site.

**Conclusion:**

In children aged 6–18 months living in malaria-endemic settings, LAZ, WAZ, and WLZ do not predict malaria incidence. However, WLZ may be associated with prevalence of malaria. Socio-economic and micro-geographic factors may explain the variations in malaria, but these require further study.

*Trial registration* NCT00945698. Registered July 24, 2009, https://clinicaltrials.gov/ct2/show/NCT00945698, NCT01239693. Registered Nov 11, 2010, https://clinicaltrials.gov/ct2/show/NCT01239693

## Background

Malaria is one of the most serious public health problems in the world, with an estimated 216 million cases and 445,000 deaths reported in 2016, and approximately 90% of the cases and deaths occurring in the African Region [1]. Malawi is one of the malaria-hyperendemic countries in sub-Saharan Africa, with around 4 million malaria cases reported annually [2]. In malaria-hyperendemic countries, it is assumed that virtually all exposed individuals would suffer a malaria episode by early childhood [3]. However, several studies have shown that in these settings, only a small proportion of children suffer repeated malaria infections, and these children are responsible for most of the malaria cases [4–6], an over-dispersion known as the ‘20/80’ rule [7].

The suggested underlying factors for this over-dispersion in malaria infections are varied, and include for instance genetic [5], behavioural [8] and environmental factors [6]. Other studies suggest that undernutrition plays an important role in malaria epidemiology because of the synergistic interactions between nutrition and infections [9–11]. However, several reviews on the relationship between undernutrition and malaria determined that the current evidence is inconclusive; attributed to the heterogeneity in the study populations, malaria parasite species, and host-parasite relationship [12–14]. Hence the need for further understanding of the role of undernutrition in malaria epidemiology.

The International Lipid-based Nutrient Supplements (iLiNS) Project DOSE and DYAD studies were randomized controlled trials conducted in Malawi to study the impact of lipid-based nutrient supplements (LNS) on growth of children [15, 16]. Analysis of longitudinal malaria data in the two studies showed that 39% of the infants and young children aged 6 to 18 months did not report any malaria episode in the one-year study period; only 30.7% reported more than one episode of ‘presumed’ malaria but these were responsible for 73.7% of the ‘presumed’ malaria episodes [17]. The aim of the current analysis was to further investigate the distribution of malaria in children in these cohorts and determine whether this distribution is predicted by anthropometric status at 6 months. The hypothesis was that lower length for age z-scores (LAZ), weight-for-age z-scores (WAZ), and lower weight for length z-scores (WLZ) at 6 months will be associated with a higher incidence of malaria from 6 to 18 months, and higher prevalence of malaria at 18 months.

## Methods

### Study setting

The iLiNS-DOSE and iLiNS-DYAD-M studies were conducted in four facilities: one public district hospital (Mangochi), one mission hospital (Malindi), and two rural public health centres (Lungwena and Namwera) in Mangochi District, Southern Malawi. The total catchment population of 180,000 largely subsisted on farming and fishing. Mangochi site is low-lying at an altitude of ~ 485 m above sea level, but traversed by the Shire River (the largest river in Malawi). Two of the study sites (Lungwena and Malindi) are also low-lying with the similar altitude along the eastern shore of Lake Malawi. In contrast, Namwera lies at the top of Namwera Hills, bordering Mozambique, at an altitude of ~ 900 m above sea level and is far from the large water bodies. Namwera experienced higher rainfall and cooler temperatures than the other three study sites [18, 19].

In Malawian children aged < 5 years, the prevalence of malaria (by microscopy), diarrhoea and acute respiratory infections were 24.3%, 22% and 5%, respectively, with seasonal fluctuations [2, 20]. The sub-tropical climate comprising a warm, wet season from November to April, a cool, dry winter season from May to August, and a hot, dry season from September to October [21] is favourable for the *Anopheles* mosquitoes which transmit *Plasmodium* parasites. *Plasmodium falciparum* is the most dominant and causes about 98% of all malaria infections in Malawi. Malaria transmission occurs throughout the year with highest transmission rates occurring between October and April (rainy season), mainly in low-lying and high temperature areas.

### Study design, data collection and ethics statement

The data for this analysis were taken from the iLiNS-DOSE and iLiNS-DYAD-M studies—two large community-based randomized controlled trials conducted in rural Malawi.

In the iLiNS-DOSE study, 6-months old children were randomly allocated to one of five intervention groups provided with different doses or formulations of LNS or to a control group that did not receive LNS during the 12-month study period, between November 2009 and May 2012. In the iLiNS-DYAD-M study, pregnant women < 20 weeks’ gestation were randomly allocated to one of three groups to receive iron and folic acid (IFA), multiple micronutrients (MMN) or a small-quantity (20 g) of LNS daily. After delivery, women in the IFA group received placebo tablets, while MMN and LNS supplementation was continued up to 6 months postpartum. Children of mothers in the LNS group also received LNS 10 g twice daily from 6 to 18 months. This study was conducted from February 2011 to April 2015. Details of design, randomization and enrolment for the two studies can be found in the main outcome papers [15, 16].

In both studies, research assistants visited the children’s homes every week from age 6 to 18 months to interview the guardians about the child’s health in the previous 7 days using a structured questionnaire. The information was complemented by a picture calendar filled out by the guardians daily to aid memory of their children’s morbidity status. These were done to minimise problems of recall associated with community morbidity assessments [22, 23]. The use of maternal interviews to collect data on child morbidity has been validated in previous studies [24, 25]. The research assistants referred all cases of fever to the nearby health facility for a malaria rapid diagnostic test (RDT) and treatment with artemether/lumefantrine, the nationally recommended anti-malarial drug. The children were followed throughout the year, covering periods of both high and low malaria transmission.

Facility health workers were trained to collect data on clinical diagnosis, RDT, and/or malaria microscopy results whenever the child was treated at the health facility. In addition, all children had malaria microscopy tests done during the scheduled study clinic visits at age 6 months and 18 months. Blood smears were obtained from all children at the time of the blood sampling for biochemical assessments, stained with 2% Giemsa for 30 min. All thick slides were reviewed by two microscopists using a high-power microscope to determine the presence of malaria parasites. Discrepant readings were resolved by a third reviewer. Malaria RDT was also done at 6 months scheduled clinic visit using rapid test kit (Clearview Malarial Combo, Alere, South Africa).

Anthropometric assessments were done at 6 months and 18 months. Study anthropometrists measured the infant’s length with a high-quality length board (Harpenden Infantometer; Holtain Limited) and recorded it to the nearest 1 mm. They weighed unclothed infants with electronic infant weighing scale (SECA 735; Seca GmbH & Co), recording to the nearest 10 g. The anthropometrists were trained and their measurement reliability was verified at the start of the study and at 6-months intervals thereafter with methods adapted from the procedures used in the WHO Multicentre Growth Reference Study [26]. The anthropometrists calibrated all equipment with standard weights and length rods daily.

Study nurses collected 5–7 mL of blood by venepuncture using a 23-gauge needle into 7.5 mL evacuated, trace element-free polyethylene tubes containing lithium heparin (Sarstedt Monovette, NH4‐heparin, Sarstedt Inc., Newton, NC, USA). The blood tube was immediately inverted 10 times to mix the heparin anticoagulant with the blood to prevent clotting. A small aliquot of the whole blood was pipetted out and used to analyse haemoglobin (Hb) on the Hemocue 201+ system (Hemocue, Brea, CA, USA). The tube containing the remaining whole blood was then placed in an insulated cooler with ice packs and processed within 2 h of collection. Trained laboratory staff then aliquoted whole blood into microcuvettes and measured zinc protoporphyrin (ZPP) concentration from unwashed venous blood sample using a haematofluorometer (206D, AVIV Biomedical Inc., Lakewood, NJ, USA).

At enrolment, research assistants interviewed the mothers to obtain household level information including assets, number of children, maternal education (years spent in school) and maternal age. Household food security was assessed using Household Food Insecurity Access Scale (HFIAS) [27]. Use of insecticide treated bed nets (ITNs) was assessed by asking the guardians the number of days that the child slept under a bed net during the week preceding the interview at 6 months. Site was defined based on the clinics where the two studies were conducted (i.e. Namwera, Mangochi, Malindi, and Lungwena).

### Definition of the predictors and the outcomes

#### Outcome variables

The primary outcome for this analysis was the incidence of ‘presumed’ malaria, derived from the weekly morbidity data. ‘Presumed’ malaria diagnosis was defined as history of fever either reported by the guardians or tympanic temperature ≥ 38 °C measured by the research assistants during the home visit. An episode of ‘presumed’ malaria was defined as the period starting from the day the child had malaria symptoms, preceded by at least 2 days of no symptoms or no data. The episode ended on the last day the child had malaria symptoms, if followed by at least 2 symptom-free days. Fever episodes accompanied by respiratory signs or diarrhoea were excluded from the malaria diagnosis.

Secondary outcomes included (1) incidence of clinical malaria, taken from the malaria diagnosis made by the health worker in the absence of a diagnostic test whenever the child visited a health facility, (2) incidence of confirmed malaria, taken from the malaria diagnosis made by the health worker confirmed by a positive RDT, and (3) prevalence of malaria parasitaemia derived from the malaria microscopy results at age 18 months. Malaria incidence was calculated as total episodes of malaria/total child years at risk; malaria parasitaemia prevalence was calculated as the proportion with a positive malaria parasite slide.

#### Predictor variables

Length for age z-scores (LAZ), weight-for-age z-scores (WAZ), and weight for length z-scores (WLZ) were calculated from the anthropometry data using WHO growth standards [26]. Iron deficiency at age 6 months was defined as whole blood ZPP > 70 µmol/mol haem [28]. HFIAS z-scores were generated by summing the value of responses to nine questions regarding food insecurity: the higher the score, the higher degree of food insecurity in the last 4 weeks [27]. Household asset scores were defined as the principal components score based on baseline ownership of a set of assets and household quality: the higher the score, the better the living conditions. Number of children aged < 5 years was defined as number of children below the age of five who were part of the participant’s household at 6 months.

### Statistical analysis

All children who had malaria data at any point from age 6 to 18 months were included in the analysis. Negative binomial regression was used to assess the association of LAZ, WAZ, and WLZ (independent variables) with the incidence of malaria (dependent variable), and Poisson regression (with a robust variance estimator) [29] to assess the association of LAZ, WAZ, and WLZ with prevalence of malaria parasitaemia.

To study the independent effect of various predictors, multivariate regression models were constructed that included potential predictors collected at 6 months. The following were potential predictors: household asset scores, maternal age (centred around the mean), and education, number of children aged < 5 years, HFIA score, whether the child received the study intervention (LNS) or not, adjusted for the additional control group [MMN] in iLiNS-DYAD, sex of the child, Hb concentration, iron status, month of birth, daily use of ITNs, distance to health facility and study site. Collinearity among the predictors was tested using the collin stata command. If the predictors were highly collinear (> 0.5), the one that was less strongly associated with the outcomes was dropped.

The results are reported as incidence rate ratios [IRR] or prevalence ratio [PR] and their 95% confidence intervals (95% CI) at p = 0.05. Robust standard errors were computed to adjust for correlation of recurrent malaria episodes in a single child.

Other potential predictors were considered including immunization status, markers of inflammation (C-reactive protein and alpha1-acid glycoprotein concentrations) at 6 months, maternal and child malaria immunity, and maternal HIV status. However, these variables were available only from a subsample of the two studies, hence were eventually dropped from the final models to maximize the sample size. Furthermore, these variables showed little effect on the model during sensitivity analysis. Stata version 14 (StataCorp, Texas, USA) was used for the main analyses.

## Results

### Study population

**Fig. 1:**
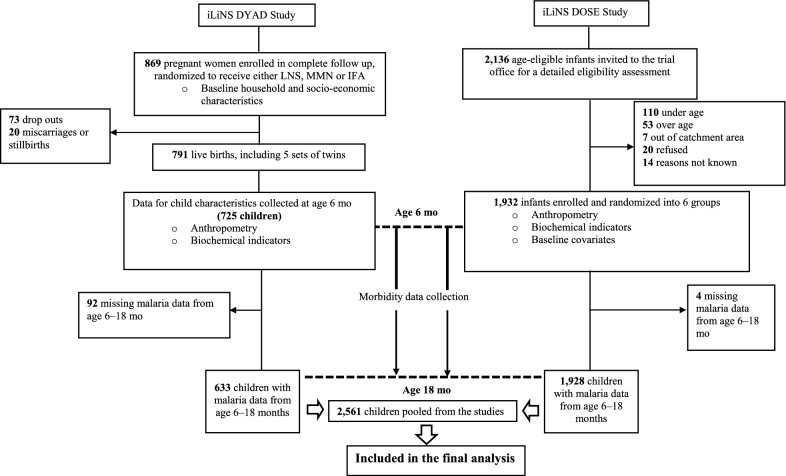
Flow chart of the children enrolled and included in the final analysis. The figure shows the number of children enrolled, children lost to follow up, and children who were eventually included in the analysis from the iLiNS DOSE and iLiNS DYAD-M cohorts

Of the 2723 children enrolled in the two study cohorts, 2561 (94%) had data for the primary outcome (1928 children from the iLiNS DOSE study and 633 children from the iLiNS DYAD-M study). These were included in the final analysis (Fig. 1). At age 6 months, the mean (SD) length-for-age z-scores (LAZ), weight-for-age z-scores (WAZ), and weight-for-length z-scores (WLZ) were − 1.4 (1.1), − 0.7 (1.2), and 0.3 (1.1) respectively. The proportions of children who were stunted, underweight and wasted were 28.3%, 12.9%, and 2.0%, respectively. Further characteristics of these children at age 6 months are summarized in Table 1.

**Table 1:** Participant characteristics at age 6 months

	Mean (SD) or n (%)	N
*Child factors*		
Mean (SD) weight-for-length z-score	0.3 (1.1)	2561
Mean (SD) weight-for-age z-score	− 0.7 (1.2)	2561
Mean (SD) length-for-age z-score	− 1.4 (1.1)	2561
Mean (SD) haemoglobin, g/L	104 (16)	2523
Proportion of boys	1265 (49.4%)	2561
Proportion wasted	50 (2.0%)	2561
Proportion underweight	330 (12.9%)	2561
Proportion stunted	725 (28.3%)	2561
Proportion with haemoglobin < 105 g/L	1334 (51.5%)	2523
Proportion with ZPP > 70 µmol/mole haem^a^	1567 (66.1%)	2371
Proportion with malaria positive by RDT	349 (14.6%)	2389
*Maternal factors*		
Mean (SD) maternal age (years)	25.8 (6.2)	2265
Mean (SD) maternal education, completed years	4.4 (3.6)	2425
*Socio*-*economic and household factors*		
Mean (SD) Household Food Insecurity Access Score	6.1 (5.6)	2128
Mean (SD) asset score	− 0.02 (0.99)	2179
Mean (SD) number of children aged < 5 years in the household	1.6 (0.7)	2149
Proportion who slept under ITN daily^b^	2065 (81.1%)	2546
*Environmental factors*		
Proportion who received LNS intervention	1804 (70.6%)	2556
*Study site*		
Mangochi	1667 (65.2%)	2561
Namwera	445 (17.4%)	2561
Malindi	120 (4.7%)	2561
Lungwena	324 (12.7%)	2561

ITN, insecticide-treated bed nets; LNS, lipid-based nutrient supplement; RDT, malaria antigen rapid diagnostic test; ZPP, zinc protoporphyrin

^a^Measured from unwashed venous blood

^b^A week prior to the day of assessment

### Incidence and prevalence of malaria

The children contributed 2405.6 child years of follow up, i.e. the mean (SD) duration of follow up was 344 (73) days/child. During the home visits, 27,340 morbidity episodes were reported, 9.3% (2549/27,340) of which were episodes of ‘presumed’ malaria. The rest were due to: ARI, 53.8% (14,708/27,340); diarrhoea, 23% (6282/27,340) and minor conditions, 13.9% (3801/27,340).

Of the total morbidity episodes identified at the home visits, 44% (12,048/27,340) were reported and treated at a health facility, 32.5% (3917/12,048) of which were treated for malaria, 76.8% (3007/3917) of them confirmed by RDT.

From the home visits data, the mean (SD) incidence of all illnesses combined was 16.2 (13.1) episodes per child year. The mean (SD) incidence of ‘presumed’ malaria was 1.1 (1.6) episodes per child year. The mean (SD) incidence of acute respiratory infections was 7.8 (8.0) episodes per child year and the mean (SD) incidence of diarrhoea was 2.7 (2.7) episodes per child year. Conversely, the national data show that diarrhoea incidence is higher than ARI incidence in children under-five, probably because the age ranges are slightly different. The national data do not provide specific incidences for children under 18 months.

From the hospital visits data, the mean (SD) incidences of clinical malaria and confirmed malaria were, respectively, 0.4 (0.8) and 1.3 (2.0) episodes per child year (i.e. the mean (SD) incidence of all malaria at hospital visits was 1.7 (2.4) episodes per child year).

The prevalence of malaria parasitaemia (by microscopy) increased from 4.8% at age 6 months to 9.6% at age 18 months. During the 12-month follow up period, 45.1% (1156/2561) of the children included in this analysis did not report any episode of malaria, 28.4% (728/2561) reported one episode and 26.4% (677/2561) reported > 1 malaria episodes. The children who reported > 1 malaria episodes were responsible for 71.4% (1821/2549) of all malaria episodes reported in the two studies. These findings were similar across the different definitions of malaria.

### Association of LAZ at 6 months with malaria from 6 to 18 months

In bivariate analysis, a higher LAZ at 6 months was associated with a lower incidence of clinical malaria and lower incidence of confirmed malaria from 6 to 18 months (Table 2, unadjusted), and lower prevalence of malaria parasitaemia at 18 months (Table 3, unadjusted), but not incidence of ‘presumed’ malaria (Table 2, unadjusted). When adjusted for other predictors, these associations were no longer evident (Tables 2, 3, adjusted, and 4).

**Table 2:** Association of length-for-age, weight-for-length, and weight-for-age z-scores at age 6 months with malaria incidence from age 6 months to 18 months

	Incidence of ‘presumed’ malaria (N = 2561)				Incidence of clinical malaria (N = 2497)				Incidence of confirmed malaria (N = 2497)			
	Unadjusted		Adjusted^a^		Unadjusted		Adjusted^a^		Unadjusted		Adjusted^a^	
	IRR^b^ (95% CI)	p-value	IRR^b^ (95% CI)	p-value	IRR^b^ (95% CI)	p-value	IRR^b^ (95% CI)	p-value	IRR^b^ (95% CI)	p-value	IRR^b^ (95% CI)	p-value
*Predictor at age 6* *months*												
LAZ	0.98 (0.94 to 1.02)	0.370	1.03 (0.98 to 1.09)	0.394	0.91 (0.85 to 0.98)	0.011	0.98 (0.89 to 1.07)	0.703	0.92 (0.87 to 0.97)	0.003	1.00 (0.94 to 1.07)	0.882
WLZ	0.96 (0.92 to 1.01)	0.098	0.97 (0.92 to 1.02)	0.190	0.99 (0.95 to 1.04)	0.762	1.03 (0.94 to 1.12)	0.571	0.99 (0.94 to 1.04)	0.623	1.00 (0.94 to 1.06)	0.950
WAZ	0.97 (0.93 to 1.01)	0.094	1.10 (0.50 to 2.42)	0.810	0.95 (0.88 to 1.01)	0.093	1.07 (0.29 to 3.91)	0.916	0.95 (0.90 to 0.99)	0.030	1.46 (0.48 to 4.40)	0.506

CI: confidence interval; IRR: incidence rate ratio; LAZ: length-for-age z-score; WAZ: weight-for-age z-score; WLZ: weight-for-length z-score

^a^Adjusted for the following factors at age 6 months: sex of the child, haemoglobin concentration, iron status, month of birth, daily use of insecticide-treated bed nets, distance to health facility, study site, asset scores, maternal age, maternal education, children < 5 years, HFIA score, and whether the child received the study intervention (LNS) or not

^b^Incidence rate ratio, obtained using negative binomial regression. The IRR represents the rate of change in incidence of malaria for each 1-SD higher LAZ, WAZ, or WLZ

**Table 3:** Association of length-for-age, weight-for-length, and weight-for-age z-scores at age 6 months with prevalence of malaria parasitaemia at age 18 months

	Prevalence of malaria parasitaemia at age 18 months (N = 1662)^a^			
	Unadjusted		Adjusted^b^	
	PR^c^ (95% CI)	p-value	PR^c^ (95% CI)	p-value
*Predictor at age 6* *months*				
LAZ	0.82 (0.72 to 0.93)	0.003	1.11 (0.93 to 1.33)	0.259
WLZ	1.01 (0.88 to 1.16)	0.877	0.80 (0.67 to 0.94)	0.007
WAZ	0.85 (0.75 to 0.96)	0.007	0.88 (0.75 to 1.03)	0.115

CI: confidence interval; LAZ: length-for-age z-score; PR: prevalence ratio; WAZ: weight-for-age z-score; WLZ: weight-for-length z-score

^a^Positive result from malaria microscopy readings

^b^Adjusted for the following factors at age 6 months: sex of the child, haemoglobin concentration, iron status, month of birth, daily use of insecticide-treated bed nets, distance to health facility, study site, asset scores, maternal age, maternal education, children < 5 years, HFIA score, and whether the child received the study intervention (LNS) or not

^c^Prevalence ratio, obtained using a modified Poisson regression (with a robust variance estimator) [29]. The PR represents the rate of change in prevalence of malaria parasitaemia for each 1-SD higher LAZ, WAZ, or WLZ

**Table 4:** Independent predictors of incidence and prevalence of malaria in multivariate analysis

	Incidence of ‘presumed’ malaria (N = 2561)		Incidence of clinical malaria (N = 2497)		Incidence of confirmed malaria (N = 2497)		Prevalence of malaria parasitaemia at age 18 months (N = 1662)	
	IRR^a^ (95% CI)	p-value	IRR^a^ (95% CI)	p-value	IRR^a^ (95% CI)	p-value	PR^b^ (95% CI)	p-value
*Child factors at age 6* *months*								
Haemoglobin (g/L)	1.00 (0.99 to 1.01)	0.529	0.99 (0.98 to 1.00)	0.016	1.00 (0.99 to 1.01)	0.845	0.98 (0.97 to 0.99)	0.001
Iron deficiency^c^	1.04 (0.91 to 1.18)	0.587	1.03 (0.83 to 1.28)	0.773	1.20 (1.04 to 1.39)	0.012	2.11 (1.24 to 3.57)	0.006
*Maternal factors at enrollment*								
Maternal age	1.01 (1.00 to 1.02)	0.011	0.99 (0.97 to 1.01)	0.149	1.00 (0.98 to 1.01)	0.200	0.97 (0.94 to 0.99)	0.033
Maternal education	0.99 (0.97 to 1.01)	0.427	1.00 (0.98 to 1.03)	0.783	0.97 (0.95 to 0.99)	0.024	0.90 (0.85 to 0.96)	0.001
*Household factors at enrollment*								
Household Food Insecurity Access Score	1.00 (0.99 to 1.01)	0.809	1.02 (1.01 to 1.04)	0.022	1.01 (0.99 to 1.02)	0.065	1.02 (0.99 to 1.05)	0.184
Asset scores	0.86 (0.80 to 0.93)	< 0.001	0.94 (0.83 to 1.07)	0.343	0.73 (0.64 to 0.83)	< 0.001	0.92 (0.63 to 1.34)	0.666
*Environmental factors*								
LNS intervention (vs control)	1.00 (0.88 to 1.14)	0.983	1.11 (0.89 to 1.37)	0.343	1.02 (0.86 to 1.22)	0.783	1.07 (0.70 to 1.62)	0.765
*Study site*								
Mangochi	Reference		Reference		Reference		Reference	
Namwera	0.94 (0.81 to 1.09)	0.457	1.49 (1.20 to 1.85)	< 0.001	1.73 (1.50 to 1.99)	< 0.001	1.49 (0.99 to 2.24)	0.054
Malindi	1.12 (0.88 to 1.42)	0.337	0.45 (0.27 to 0.73)	0.001	0.58 (0.43 to 0.78)	< 0.001	0.74 (0.29 to 1.88)	0.531
Lungwena	0.91 (0.75 to 1.09)	0.303	0.24 (0.14 to 0.41)	< 0.001	0.33 (0.25 to 0.44)	< 0.001	0.65 (0.36 to 1.19)	0.163

Other factors included in the models but not significant were: sex of the child, birth month, number of children in household, and daily use of insecticide-treated bed nets

CI: confidence interval; IRR: incidence rate ratio; LNS: lipid-based nutrient supplements; PR: prevalence ratio; ZPP: zinc protoporphyrin

^a^ Incidence rate ratio, obtained using negative binomial regression. The IRR represents the rate of change in incidence of malaria (for each 1-unit higher in the continuous predictors or for each group compared to the reference group in the categorical predictors), adjusted for other variables

^b^ Prevalence ratio, obtained using a modified Poisson regression (with a robust variance estimator) [29]. The PR represents the rate of change in prevalence of malaria parasitaemia (for each 1-unit higher in the continuous predictors or for each group compared to the reference group in the categorical predictors), adjusted for other variables

^c^ ZPP > 70 µmol/mol heme [28]

### Association of WLZ at 6 months with malaria from 6 to 18 months

There was no association between WLZ at 6 months and the incidence of ‘presumed’ malaria, clinical malaria or confirmed malaria from 6 to 18 months, in either bivariate or multivariate analyses (Table 2). In multivariate analysis, higher WLZ at 6 months was associated with lower prevalence of malaria parasitaemia at 18 months, adjusted for other predictors (i.e. 1 SD higher WLZ at 6 months was associated with 20% decrease in prevalence of malaria parasitaemia at 18 months) (Tables 3, 4).

### Association of WAZ at 6 months with malaria from 6 to 18 months

In bivariate analysis, a higher WAZ at 6 months was associated with lower incidence of confirmed malaria from 6 to 18 months (Table 2, unadjusted), and lower prevalence of malaria parasitaemia at 18 months (Tables 3, unadjusted, 4), but not incidence of ‘presumed’ malaria nor incidence of clinical malaria (Table 2, unadjusted). When adjusted for other predictors, these associations were no longer evident (Tables 2, 3, adjusted and 4).

### Independent predictors of malaria

Independent predictors that were significantly associated with malaria outcomes are presented in Tables 3, 4. Incidence of ‘presumed’ malaria from 6 to 18 months was independently associated with maternal age and asset score at 6 months (i.e. each year higher in maternal age was associated with 1% increase in incidence of ‘presumed’ malaria; and 1 SD higher asset score was associated with 14% decrease in incidence of ‘presumed’ malaria).

Incidence of clinical malaria from 6 to 18 months was independently associated with Hb concentration and HFIAS at 6 months (i.e. each g/L higher Hb at 6 months was associated with 1% decrease in incidence of clinical malaria; and 1-unit higher HFIAS was associated with 2% increase in incidence of clinical malaria). Higher HFIAS represents higher degree of food insecurity. There was also a significant variation in the incidence of clinical malaria between the health facilities (study sites), with Namwera site reporting higher incidence of clinical malaria compared to Mangochi, while the other two sites (Malindi and Lungwena) had lower incidence of clinical malaria compared to Mangochi.

Incidence of confirmed malaria from 6 to 18 months was independently associated with iron deficiency, maternal education and asset score at 6 months (i.e. iron deficiency at 6 months was associated with 20% higher incidence of confirmed malaria; each year higher in maternal schooling was associated with 3% decrease in incidence of confirmed malaria; 1 SD higher asset score was associated with 27% decrease in incidence of confirmed malaria). There was also a significant variation in the incidence of confirmed malaria between the study sites with Namwera site reporting higher incidence of confirmed malaria compared to Mangochi, while the other two sites (Malindi and Lungwena) reported lower incidence of confirmed malaria compared to Mangochi.

Independent predictors of malaria parasitaemia prevalence were WLZ, maternal age, maternal education, Hb concentration, and iron deficiency (i.e. 1 SD higher WLZ was associated with 20% decrease in prevalence of malaria parasitaemia (see Tables 3, 4); each year higher in maternal age was associated with 3% decrease in prevalence of malaria parasitaemia; each additional year in maternal schooling was associated with 10% decrease in prevalence of malaria parasitaemia; each g/L higher Hb at 6 months was associated with 2% decrease in prevalence of malaria parasitaemia; and iron deficiency at 6 months was associated with 111% higher prevalence of malaria parasitaemia).

## Discussion

The study hypothesis was that lower length for age z-scores (LAZ) and lower weight for length z-scores (WLZ) at 6 months will be associated with a higher incidence of malaria from 6 to 18 months and higher prevalence of malaria parasitaemia at 18 months. In a sample of 2561 Malawian children, LAZ at 6 months was not associated with incidence of malaria from 6 to 18 months, nor prevalence of malaria parasitaemia at 18 months. Higher WLZ at 6 months was associated with lower prevalence of malaria parasitaemia at 18 months (20% decrease in prevalence of malaria parasitaemia at 18 months for every 1 SD higher WLZ at 6 months), but not with incidence of malaria from 6 to 18 months.

The longitudinal study design helped to determine the temporality of the associations because (1) malaria outcomes were obtained from 6 to 18 months after identifying the predictors at 6 months, and (2) malaria is less common in children < 6 months due to the protective effect of fetal haemoglobin and maternal antibodies [30–32]. Another strength of the study was the enhancement of morbidity recall using a daily pictorial calendar which minimized recall bias associated with home morbidity assessments [22, 23]. The study had a long follow up which allowed assessment of the associations in all seasons, and by pooling data from two large cohorts we ended up with a large sample size with adequate power to detect associations.

The main weakness of the study is the change in the exposure (anthropometric status) during the follow up. There was significant decrease in mean LAZ, WAZ, and WLZ from 6 months to 18 months. Compared to baseline, more children had developed stunting, underweight, and wasting by 18 months (41.5% vs 28.3%, 16.5% vs 12.9%, and 5% vs 2% respectively), indicating that some children became nutritionally worse off during follow up which may have led to misclassification bias.

There is possibility of residual confounding due to unmeasured factors associated with malaria including HIV infection [33], polyparasitism [34], vitamin A and zinc deficiency [35–38], and genetic variations including haemoglobinopathies [39–41].

Presumptive diagnosis of malaria may also have led to overestimating the outcome whereas the use of RDT-confirmed malaria may have led to under-estimating the outcome because (1) 23% of the children were treated for malaria at the hospital without RDT confirmation, and (2) children who had malaria episodes but received home treatment or no treatment and did not present to the hospitals were missed (only 44% of the morbidity episodes were treated at the hospital). However, the consistency of the results across the different malaria definitions suggests that this weakness did not bias the results, and considering the strengths of this study, the conclusions are reasonably valid.

The finding of no association between stuntedness at 6 months and malaria in the subsequent 12-month period is similar to results of previous studies [42–45], which suggests that stunting may be of little significance in malaria epidemiology. These results are however different from those of other studies that reported an increased risk of malaria associated with stunting. In the Gambia, a prospective study of children aged < 5 years reported an increased risk of malaria associated with stunting (crude RR = 1.35; 95% CI 1.08–1.69) [9]. The follow up was very short (during the malaria season that lasted only 20 weeks), and although the outcomes were adjusted for age, sex, and ethnicity, crude RR were reported. The authors also did not adjust for socio-economic and household factors therefore could not rule out the influence of these confounders in their results. In Kenya, stunting in children aged 0–36 months was associated with an increased odds of malaria parasitaemia (odds ratio = 1.98, p < 0.0001) [10]. The cross-sectional design of this study suggests the observed association may have been due to reverse causation, i.e. the cumulative deleterious effects of malaria on linear growth. In Uganda, mild stunting (IRR = 1.24, 95% CI 1.06–1.46) and moderate-severe stunting (IRR = 1.24, 95% CI 1.03–1.48) in children aged < 2.5 years were associated with increased incidence of malaria parasitaemia [11]. However, although this was a cohort study, it was not clear whether exposure assessment preceded the outcomes, therefore, the temporal relationship between stunting and risk of malaria was difficult to ascertain.

However, a study in Papua New Guinea reported that lower LAZ was associated with lower incidence of malaria in the subsequent one-year period, attributed to increased interferon γ (IFN-γ) response to specific malarial antigens observed in stunted children, although the mechanisms were not fully explained [46]. Malaria varies with age (incidence of malaria decreases whereas prevalence of malaria parasitaemia increases with age) [13, 47, 48], therefore, the wide age range of the study participants (from 10 months to 10 years) makes for difficult comparisons between the studies. In the Papua New Guinea study, the authors noted that interferon-γ release increased with age, the prevalence rates of splenomegaly and parasitaemia increased with age, whereas the incidence of malaria decreased with age, further muddling the interpretation of the findings.

In this population, supplementation with LNS did not alter malaria antibody acquisition [49], which was attributed to the finding that the nutritional supplements also did not promote infant growth [16]. Further analysis showed no difference in malaria antibody acquisition between the stunted and non-stunted children at 6 months (data not shown), supporting the null findings.

In this study, higher WLZ at 6 months was associated with lower prevalence of malaria parasitaemia at 18 months, when adjusted for other predictors. Wasting is associated with low levels of leptin through depletion of fat mass [50]. Low levels of leptin can lead to reduced immune response [51], resulting in increased risk of malaria associated with wasting. So far, the evidence of association (increased or decreased risk) of malaria with WLZ has mainly come from cross-sectional studies [10, 52, 53]. A recent systematic review determined that the relationship between wasting and risk of malaria is hitherto inconclusive with most longitudinal studies reporting no association [54]. This association was not conclusive and may be due to chance (e.g., because of multiple testing [55]), considering that WLZ was significantly associated with prevalence of malaria parasitaemia only and not with the other malaria outcomes.

In this study, only 30% of the children reported frequent malaria episodes. Knowledge of this over-dispersion in malaria infections is important because interventions targeted at this core group could be the most effective [7]. However, providing routine screening to identify such children is difficult. Platforms such as growth monitoring and promotion clinics (GMP) provide an entry point to preventive and curative health care and have been associated with significant reductions in malnutrition and mortality [56].

Such platforms can be useful for malaria screening especially in malaria hypoendemic or mesoendemic settings where targeting malaria interventions to undernourished children has potential to reduce malaria morbidity and mortality [57]. In this setting, socio-economic factors such as household asset scores, maternal education, and food insecurity were significantly associated with malaria. There were also variations in hospital-diagnosed malaria by study site, with the Namwera site reporting higher incidences of clinical and RDT-confirmed malaria compared to the other three study sites. These factors may be more important than anthropometric status in malaria epidemiology but were not explored further in this analysis.

## Conclusions

In conclusion, in children aged 6 to 18 months living in this malaria-endemic setting, LAZ, WAZ, and WLZ do not predict subsequent malaria incidence when adjusted for other predictors. However, WLZ may be associated with prevalence of malaria parasitaemia. Socio-economic factors and micro-geographic variations may explain most of the over-dispersion in malaria infections in this setting, but these require further study.

## Abbreviations

CI: confidence interval
GMP: growth monitoring and promotion
Hb: hemoglobin
HFIA: household food insecurity access
HIV: human immuno-deficiency virus
IFA: iron and folic acid
IMCI: integrated management of childhood illness
iLiNS: international lipid-based nutrient supplements project
ITNs: insecticide-treated bed nets
LAZ: length-for-age z-scores
LNS: lipid-based nutrient supplements
MMN: multiple micronutrients
RDT: malaria rapid diagnostic test
PR: prevalence ratio
SD: standard deviation
WAZ: weight-for-age z-scores
WHO: World Health Organization
WLZ: weight-for-length z-scores
ZPP: zinc protoporphyrin
